# Which factors influence the oral health of nursing-home residents with cognitive and motor impairments?

**DOI:** 10.1007/s40520-020-01503-5

**Published:** 2020-03-06

**Authors:** Anna-Luisa Klotz, Melania Zajac, Judith Ehret, Samuel Kilian, Peter Rammelsberg, Andreas Zenthöfer

**Affiliations:** 1 Department of Prosthetic Dentistry, University of Heidelberg, Dental School, Im Neuenheimer Feld 400, 69120 Heidelberg, Germany; 2 Institute of Medical Biometry and Informatics, University of Heidelberg, Im Neuenheimer Feld 130.3, 69120 Heidelberg, Germany

**Keywords:** Oral health, Oral hygiene, Older people, Motor and cognitive impairment, Nursing home

## Abstract

**Background:**

There is limited information available about the oral and denture hygiene and oral health of nursing-home residents with cognitive and motor impairments.

**Aims:**

The purpose of this study was to identify factors influencing the oral and denture hygiene and oral health of nursing-home residents with cognitive and motor impairments.

**Methods:**

The study was performed in nine nursing-homes in Germany. Sociodemographic and general data were collected for all participants (*n* = 150). The Clinical Dementia Rating (CDR) was used to identify the presence of dementia, and the Apraxia Screening Test (AST) was used to identify motor impairment. A comprehensive dental examination was also performed. This included the documentation of dental and denture status and the number of decayed, missing and filled teeth (DMFT). In addition, dental and denture hygiene were assessed using the Plaque Index (PI) and the Denture Hygiene Index (DHI). Univariate and multivariate regression models were used to analyse possible factors affecting the dependent target variables.

**Results:**

In multivariate regression analysis, the factors that most strongly influenced greater PI were a lower number of medications taken (*p* = 0.018), poorer general health (*p* = 0.013) and the presence of dementia (*p* < 0.010). A more advanced age (*p* = 0.036) and longer nursing-home stay (*p* = 0.048) had a negative effect on the DHI. Furthermore, gender (*p* = 0.037, in favour of women), poorer general health (*p* = 0.003), presence of dementia (*p* = 0.003), and the absence of natural teeth (*p* = 0.028) influenced poorer oral health. The factors most strongly influenced greater number of missing teeth were a more advanced age (*p* = 0.021) and longer nursing-home stay (*p* = 0.015). In terms of fewer filled teeth, a shorter nursing-home stay (*p* = 0.002) was the factor most strongly influenced this.

**Conclusions:**

Poorer general health and the presence of apraxia and cognitive impairment are the main determinants for poorer oral hygiene and oral health among nursing-home residents. A longer nursing-home stay also seems to be relevant for oral health and denture hygiene.

## Introduction

Ageing is a substantial risk factor for the development of general health problems, including cognitive impairment and dementia. Dementia is a common disorder among older people that becomes more prevalent with increased age [1]. In 2015, this global health problem affected an estimated 47.5 million people, a number which is expected to double by 2030 and more than triple by 2050 [2]. Many people experiencing cognitive impairment and age-related changes require professional care, and therefore move into nursing homes. Approximately 40–60% of nursing-home residents suffer from dementia [3, 4]. For this reason, both the general and oral health of older people with dementia are increasingly important research topics.

Fortunately, the oral health of self-reliant older people has improved in the last decade, and their number of remaining natural teeth has consequently increased in parallel [5, 6]. For nursing-home residents, however, the prevalence of oral-health problems such as caries, gingivitis, periodontal disease, and edentulism is still high, and inadequate oral hygiene is a major concern in long-term care [4, 7–10]. In this context, the mean number of remaining teeth among nursing-home residents is 9–12 [4, 8], and many residents urgently need dental treatment for an ulcer, infection, broken tooth or untreated caries [4, 11]. Denture-related treatment needs, too, have been described by several authors [4, 9]. Regrettably, 12–50% of nursing-home residents are edentulous, and many have no dental prosthesis at all [4, 8, 10, 12]. Such oral-health problems can be exacerbated by motor and cognitive impairments, because many older people with dementia refuse oral hygiene as part of their daily care routine. Caregivers might therefore perceive a conflict between respecting a patient’s autonomy and the provision of good care. Furthermore, as a patient’s general health worsens, less importance is often placed on their dental health [4, 13].

Several studies have found that dental health and cognition might be related in several ways [1, 12]. Nevertheless, these studies report conflicting results. On the one hand, it has been reported that oral-health problems actually become more prevalent among people with cognitive impairment and dementia [12]. Delwel et al. performed a systematic review of the oral health of people with and without dementia, and found that those with dementia had poorer oral health with more retained roots and coronal caries [12]. The number of decayed teeth has also been associated with dementia [10, 11]. In addition to these associations, it has been found that poor oral health—including tooth loss, caries and periodontal disease—might be an unrecognised risk factor for the development of dementia because of dietary changes, malnutrition and a systemic inflammatory response [1, 14]. On the other hand, no differences were found between people with and without cognitive impairment and dementia in terms of orofacial pain, number of teeth present, number of decayed, missing and filled teeth, edentulism, and the use of dentures [12].

## Aims

As far as we are aware, few studies have investigated the oral and denture hygiene and oral health of nursing-home residents with cognitive and motor impairments, yet this information would help to identify the risk factors for the oral problems mentioned above. The objective of this study was, therefore, to identify the factors associated with poorer oral and denture hygiene (using the PI and DHI) and oral health (using the DMFT) among nursing-home residents with cognitive and motor impairments.

## Methods

### Study setting

This study was approved by the local review board of the University of Heidelberg prior to its start (approval number S-420/2016). Nine long-term care facilities in Baden-Württemberg (Mannheim and Heidelberg) and Hesse [Hirschhorn (Neckar)], two of the sixteen federal states which are located in the middle and the south west of Germany, cooperated in this study. The long-term care facilities were general facilities and included each level of care. A dentist provided all residents and their legal representatives with written and oral information about the study. All residents or, if they were not *sui juris*, their legal representatives were subsequently asked to participate in the study and to give written and oral consent. To meet the inclusion criteria, residents were required to be in long-term care and have no plans to move to another institution in the next year. There were no other inclusion criteria, and 150 residents were included in the study.

### General health and cognitive status

The following information was obtained from residents’ care documentation: age (in years), gender (0 = female, 1 = male), number of diseases, regular medication, ongoing duration of nursing-home stay (in months) and level of care required. In accordance with the German care-insurance grading system, the level of care required was categorised into five grades. Residents with no care needs were classified as grade 0. Those with minor and considerable care needs were classified as grades 1 and 2, respectively. Grade 3 included residents with high care needs, whereas grade 4 included those with very high care needs. Residents with very high care needs and additional, specialised nursing requirements were classified as grade 5. The estimation of health condition of participants was also evaluated subjectively by the dentist using a three-point scale (good = 1, reduced = 2, poor = 3). In this context the estimation of participants’ constitution based on all general health variables and cognitive status. Beside these variables personal hygiene, mobility, activity, and self-sufficiency were also evaluated and included for final classification. The presence and severity of dementia were also evaluated by use of the Clinical Dementia Rating (CDR) [15]. The CDR is constructed using a five-point Likert scale and enables characterisation of six domains of cognitive and functional performance: memory, orientation, judgement, and problem solving, community affairs, home and hobbies, and personal care. Each domain was assessed by means of a structured interview with the senior and a second person (family member, care giver) [15]. Interviews were performed by two dentists trained at the memory clinic of the University of Heidelberg before the start of the study. The CDR scores were as follows: 0 = no dementia; 0.5 = very mild dementia; 1 = mild dementia; 2 = moderate dementia; 3 = severe dementia. A CDR score was recorded for each of the six domains, and the highest score from the six domains was used to calculate the final score [16].

The presence and severity of apraxia was also assessed by use of the Apraxia Screen of TULIA (AST) [17]. The AST comprises 12 items which require participants to perform tasks in the pantomime and imitation domains (five items in the pantomime domain, seven items in the imitation domain). A dichotomous scoring system is used for each item (1 = pass and 0 = fail), thus the maximum score for the AST is 12. This score is indicative of an absence of apraxia [17].

### Oral and denture hygiene and oral health

There was no uniform oral health concept in the different nursing homes and the included nursing homes put different value on the oral hygiene and health of the participants. In this context, usually the care givers decided for themselves whether and how much help the nursing home residents needed for dental care. The dental examinations of the study were performed by two dentists trained at the Department of Prosthodontics of the University of Heidelberg. Mouth mirrors and dental probes were used for the examinations.

Each participant underwent a comprehensive examination that included assessment of their dental and prosthetic status and orofacial pain. For analytical purposes, the type of prosthesis worn was categorised as follows: (1) Natural teeth or fixed dental prosthesis (FDP); (2) Removable dental prosthesis (RDP); (3) Complete denture (CD); or (4) Edentulous without replaced teeth (ENP). For the variable “total denture status”, each participant was classified according to the weaker restored jaw [18].

The quantitative plaque accumulation of natural teeth, which is indicative of oral hygiene, was also evaluated by use of the Plaque Index (PI). The PI is graded on a four-point scale (0 = no plaque to 3 = substantial plaque accumulation) before the index value is then divided by the number of surfaces assessed [19]. The Denture Hygiene Index (DHI), in which higher values indicate poorer hygiene (range 0–100%), was used to quantify denture hygiene [20]. Furthermore, decayed (D), missing (M) and filled (F) teeth were recorded by use of the DMFT Index [21]. Third molars were excluded from calculations. Scores for D, M and F could therefore range from 0 to 28 in each case [22].

### Statistical evaluation

Statistical analysis was performed by use of the software R, version 3.4.2 (R Core Team; Auckland, New Zealand). *p* values of less than 0.05 were regarded as significant. Mean values, standard deviations, absolute and relative frequencies were used to descriptively present the results. Unless otherwise stated, results are given as a mean value (± SD).

The association of participants’ characteristics with the dependent variables (PI, DHI, DMFT) was assessed by evaluating a univariate linear regression model for each covariate and each dependent variable. Furthermore, to capture the factors most strongly associated with the dependent dental variables, multivariate linear regressions were conducted for each covariate after a stepwise variable selection algorithm was performed. The stepwise variable selection algorithm firstly excluded all variables which had a *p* value greater than 0.5 in the univariate linear regressions and performed secondly a backward step, where the variable with the highest *p* value was excluded. Thirdly, it checked for every not included variable whether the *p* value, if re-included in the model, was below the threshold of 0.05. If so, the excluded variable with the smallest *p* value had been re-included (forward step). The backward and forward steps were reiterated until all included variables were significant or only one variable was left. For this, the ordinally scaled variables were dichotomised as follows: level of needed care (care level: 0 = 0–2; 1 = 3–5), general health (0 = good; 1 = reduced and poor), cognitive impairment (CDR: 0 = 0–1; 1 = 2–3) and apraxia (AST: 0 = 1–8; 1 = 9–12).

## Results

### Study population

The mean age of the study population was 82.1 (± 9.8) years, and 75.3% of the participants were female. The mean nursing-home stay among residents was 35.7 (± 33.2) months in duration. The general health of participants was evaluated as good for 81 (54.0%), reduced for 51 (34.0%) and poor for 18 (12.0%) participants. The care levels among all participants were: care level 0, four participants (2.7%); care level 1, six participants (4.0%); care level 2, 45 participants (30.0%); care level 3, 46 participants (30.7%); care level 4, 42 participants (28.0%); care level 5, 7 participants (4.7%). The mean number of diseases was 5.9 (± 3.5), and the mean number of medications taken was 8.3 (± 3.7). Most participants (87.3%) had at least mild dementia (CDR > 0). The mean value of the AST was 10.9 (± 3.0). Detailed results are given in Table 1.

**Table 1:** Descriptive statistics for participant characteristics (*n* = 150)

	Number of participants (%)	Mean (± SD)
Age	–	82.1 (± 9.8)
Gender	113 (75.3%)	
Female	37 (24.7%)	–
Male		–
Length of stay in home	–	35.7 (± 33.2)
Natural teeth		
Yes	107 (71.3%)	–
No	43 (28.7%)	–
Number of teeth	–	10.1 (± 9.8)
Level of care		
0	4 (2.7%)	–
1	6 (4.0%)	–
2	45 (30.0%)	–
3	46 (30.7%)	–
4	42 (28.0%)	–
5	7 (4.7%)	–
Number of diseases	–	5.9 (± 3.5)
Number of medications	–	8.3 (± 3.7)
Total denture status		
FDP/natural teeth	45 (30.0%)	–
RPD	27 (18.0%)	–
CD	59 (39.3%)	–
ENP	19 (12.7%)	–
Orofacial pain		
No	142 (94.7%)	–
Right	1 (0.7%)	–
Left	2 (1.3%)	–
Both sides	5 (3.3%)	–
Plaque Index (*n* = 108)	–	2.2 (± 0.9)
DHI total (*n* = 92)	–	54.4 (± 28.3)
Decayed teeth	–	1.7 (± 3.0)
Missing teeth	–	18.1 (± 9.5)
Filled teeth	–	5.2 (± 6.1)
DMFT	–	25.1 (± 4.3)
General health		
Good	81 (54.0%)	–
Reduced	51 (34.0%)	–
Poor	18 (12.0%)	–
CDR		
0	19 (12.7%)	–
0.5	37 (24.7%)	–
1	36 (24.0%)	–
2	29 (19.3%)	–
3	29 (19.3%)	–
Apraxia screening test (*n* = 144)	–	10.9 (± 3.0)

*FDP* fixed dental prosthesis, *RPD* removable partial denture, *CD* complete denture, *ENP* edentulous without replaced teeth

### Oral hygiene and health

The mean number of natural remaining teeth among the participants was 10.1 (± 9.8). Forty-five participants (30.0%) wore a fixed dental prosthesis or had natural remaining teeth; 27 (18.0%) wore a removable dental prosthesis, and 59 (39.3%) wore complete dentures in at least one jaw. Nineteen participants (12.7%) were edentulous and wore no dental prosthesis. Orofacial pain was reported by 5.3% of all participants. The mean PI, DHI and DMFT scores were 2.2 (± 0.9), 54.4 (± 28.3) and 25.1 (± 4.3), respectively. Detailed results are provided in Table 1.

### Univariate regression analysis

Univariate regression analysis detected worse PI scores among participants with the following: poorer general health (*p* < 0.001), fewer medications taken (*p* = 0.036), diagnosed apraxia (*p* = 0.017) and presence of dementia (*p* < 0.001). For DHI, none of the variables reached statistical significance, although a trend towards this could nonetheless be observed for a more advanced age (*p* = 0.051), poorer general health (*p* = 0.059) and the presence of dementia (*p* = 0.057).

Except for the presence of natural remaining teeth (*p* < 0.001), univariate analysis detected no factors affecting the number of decayed and missing teeth. However, the factors of a longer nursing-home stay (*p* = 0.002) and poorer general health (*p* = 0.031) were associated with a lower number of filled teeth. In addition, univariate analysis showed that the presence of natural remaining teeth (*p* < 0.001) associated with a higher number of filled teeth. Detailed results of the univariate regression analysis are given in Table 2.

**Table 2:** Univariate regression analysis with different dependent variables and participant characteristics

Variables	*C*	95% CI LB	95% CI UB	*R*^2^	*p* value
Plaque Index (*n* = 108)					
Age	− 0.015	− 0.032	0.002	0.029	0.079
Gender	0.262	− 0.108	0.632	0.018	0.163
Length of stay in home	0.003	− 0.003	0.008	0.009	0.316
Naturally remaining teeth^a^	− 0.682	− 1.669	0.306	0.017	0.174
Care level^a^	0.219	− 0.115	0.554	0.016	0.197
Number of diseases	0.010	− 0.035	0.055	0.002	0.665
Number of medications	− 0.049	− 0.095	− 0.003	0.041	**0.036**
General health^a^	0.582	0.270	0.893	0.114	**< 0.001**
CDR^a^	0.640	0.324	0.956	0.132	**< 0.001**
Apraxia screening test (*n* = 103)^a^	− 0.621	− 1.130	− 0.112	0.055	**0.017**
DHI (*n* = 92)					
Age	0.609	− 0.003	1.221	0.042	**0.051**
Gender	2.537	− 12.657	17.731	0.001	0.741
Length of stay in home	0.160	− 0.013	0.334	0.036	0.069
Naturally remaining teeth^a^	0.451	− 11.773	12.675	< 0.001	0.942
Care level^a^	1.633	− 10.303	13.568	0.001	0.786
Number of diseases	− 0.664	− 2.335	1.007	0.007	0.432
Number of medications	− 0.508	− 2.033	1.017	0.005	0.509
General health^a^	11.330	− 0.416	23.077	0.039	**0.059**
CDR^a^	11.571	− 0.338	23.481	0.040	**0.057**
Apraxia screening test (*n* = 89)^a^	− 0.602	− 24.088	22.884	0.000	0.959
Decayed teeth (*n* = 150)					
Age	− 0.033	− 0.083	0.016	0.012	0.186
Gender	0.381	− 0.746	1.508	0.003	0.505
Length of stay in home	0.009	− 0.006	0.024	0.010	0.225
Naturally remaining teeth^a^	2.146	1.128	3.163	0.105	< 0.001
Care level^a^	0.623	− 0.381	1.627	0.010	0.222
Number of diseases	< 0.001	− 0.141	0.141	< 0.001	0.998
Number of medications	0.009	− 0.125	0.142	< 0.001	0.898
General health^a^	0.830	− 0.136	1.797	0.019	0.092
CDR^a^	0.199	− 0.799	1.197	0.001	0.694
Apraxia screening test (*n* = 144)^a^	− 1.016	− 2.653	0.622	0.010	0.222
Missing teeth (*n* = 150)					
Age	0.152	− 0.004	0.309	0.024	0.056
Gender	− 1.442	− 5.005	2.120	0.004	0.425
Length of stay in home	0.048	0.002	0.094	0.028	0.040
Naturally remaining teeth^a^	− 12.658	− 15.371	− 9.946	0.365	< 0.001
Care level^a^	− 0.782	− 3.973	2.410	0.002	0.629
Number of diseases	0.147	− 0.298	0.591	0.003	0.516
Number of medications	0.220	− 0.200	0.641	0.007	0.302
General health^a^	1.718	− 1.357	4.794	0.008	0.271
CDR^a^	2.079	− 1.063	5.222	0.011	0.193
Apraxia screening test (*n* = 144)^a^	2.450	− 2.670	7.569	0.006	0.346
Filled teeth (*n* = 150)					
Age	− 0.032	− 0.133	0.069	0.003	0.530
Gender	1.054	− 1.226	3.333	0.006	0.363
Length of stay in home	− 0.046	− 0.075	− 0.017	0.064	0.002
Naturally remaining teeth^a^	6.847	4.973	8.721	0.261	< 0.001
Care level^a^	− 0.321	− 2.365	1.724	0.001	0.757
Number of diseases	− 0.092	− 0.377	0.193	0.003	0.524
Number of medications	− 0.153	− 0.423	0.116	0.009	0.262
General health^a^	− 2.150	− 4.096	− 0.204	0.031	0.031
CDR^a^	− 1.927	− 3.926	0.073	0.024	0.059
Apraxia screening test (*n* = 144)^a^	− 0.096	− 3.342	3.150	0.000	0.953

Significant *p* values are marked in bold

*C* regression coefficient, *LB* lower boundary, *UB* upper boundary, *R*^2^ squared Pearson correlation

^a^Participant characteristics are reported in a binary manner

### Multivariate linear regression analysis

Multivariate linear regression analysis after variable selection confirmed that the factors poorer general health (*p* = 0.013), lower number of medications taken (*p* = 0.018) and presence of dementia (*p* = 0.010) were all associated with increased plaque accumulation among participants. In contrast to the univariate linear regressions, diagnosed apraxia could not be confirmed to have a significant influence on PI scores. For DHI, the multivariate regression model after variable selection showed that a more advanced age (*p* = 0.036) and a longer nursing-home stay (*p* = 0.048) have a significant influence which could not be shown in the univariate linear regressions.

A more advanced age (*p* = 0.021) and longer nursing-home stay (*p* = 0.015) were associated with a greater number of missing teeth, whereas a shorter nursing-home stay was associated with a greater number of filled teeth (*p* = 0.002). Detailed results of the multivariate regression analysis are given in Table 3.

**Table 3:** Multivariate linear regression model after stepwise variable selection using the *p* value with different dependent variables

Variables	Coefficient	95% CI LB	95% CI UB	Std. error	*p* value
Plaque Index (*n* = 108)					
Intercept	2.223	1.827	2.620	0.120	**< 0.001**
Number of medications	− 0.051	− 0.093	− 0.010	0.021	**0.018**
General health^a^	0.420	0.092	0.750	0.165	**0.013**
CDR^a^	0.445	0.110	0.780	0.169	**0.010**
DHI (*n* = 92)					
Intercept	− 5.971	− 57.500	45.555	25.932	0.820
Age	0.648	0.044	1.251	0.304	**0.036**
Length of stay in home	0.172	0.002	0.343	0.086	**0.048**
Decayed teeth (*n* = 144)					
Intercept	2.667	1.118	4.216	0.784	**< 0.001**
Apraxia screening test^a^	− 1.016	− 2.653	0.622	0.828	0.222
Missing teeth (*n* = 150)					
Intercept	0.903	− 12.355	14.162	6.709	0.893
Age	0.184	0.029	0.340	0.079	**0.021**
Length of stay in home	0.057	0.011	0.103	0.023	**0.015**
Filled teeth (*n* = 150)					
Intercept	6.885	5.481	8.289	0.710	**< 0.001**
Length of stay in home	− 0.046	− 0.075	− 0.017	0.015	**0.002**

Significant *p* values are marked in bold

^a^Participant characteristics are reported in a binary manner

## Discussion

The results of this study suggest that poorer general health and cognitive impairment are the main factors associated with worse oral hygiene and oral health among nursing-home residents. Furthermore, the duration of the nursing-home stay appears important for both oral health and denture hygiene.

With regard to the characteristics of the study participants, it is noteworthy that the general and dental health of participants were comparable to those found in other recent studies of nursing-home residents [4, 8–10, 12, 23]. Closer inspection of the participants’ general health reveals that the number of diseases [4], number of permanently taken medications [23] and prevalence of dementia [3, 4, 10, 11] were all within the range reported for nursing-home settings. This was also true for the number of remaining natural teeth [4, 8], DMFT [8] and prevalence of edentulism [4, 8–12]. Dental and denture hygiene, as evaluated by use of the PI and DHI, were also comparably poor [4, 24]. This means that the following analysis, which has been lacking in the literature, can be generalised to a larger population.

Closer inspection of the PI revealed that the factors of poorer general health, lower number of medications taken, and presence of dementia and apraxia were strongly associated with greater plaque accumulation on teeth. In this context, the factors of number of medications taken, general health and presence of dementia were most strongly associated with greater plaque accumulation in multivariate analysis. With regard to denture hygiene, a correlative trend could be observed between poorer hygiene and a more advanced age, poorer general health and presence of dementia. However, the multivariate analysis showed that a more advanced age and longer nursing-home stay were the factors most strongly associated with poorer denture hygiene. These findings seem logical, because very elderly subjects with poor general health, motor impairment and a cognitive deficit lose the ability to adequately perform daily oral-hygiene tasks [25]. They consequently need support from caregivers, although many refuse this assistance [4, 26]. Furthermore, oral hygiene and health become comparatively less important for patients with poorer general health, and in such case are thus often not a matter of priority for caregivers. The correlation between dementia and plaque accumulation has also been described by Thomson et al. who studied 987 nursing-home residents in Canada. In addition to the presence of dementia, however, the authors also found that gender (men) and a greater number of teeth were risk factors for increased plaque accumulation [10]. In this study, conversely, gender and the presence of teeth were not found to be risk factors for PI. This difference might, however, be due to the use of different assessment tools for plaque accumulation. Another interesting finding in our study was that apraxia was not a risk factor for denture hygiene. In contrast, it does seem to be a risk factor for decayed teeth. These results are in agreement with those of Zenthöfer et al. [27]. One reason apraxia might not be a risk factor for denture hygiene is because dentures, which are cleaned outside the mouth, are more easily cleaned than natural teeth. It might also be because a very high proportion of dentures had high plaque levels [27]. Moreover, the stepwise variable selection algorithm stops if only one variable is left in the model which would explain why apraxia (not significant, see Table 3) is in the final multivariate model.

Closer inspection of dental health our study found no association between cognitive impairment and an increase in the number of decayed teeth, which is in accordance with Delwel et al. [12]. It is also worth highlighting that an absence of remaining natural teeth was associated with poorer oral health. This result shows that tooth extraction is not the best option for eliminating the oral-health problems of nursing-home residents. Another interesting finding is that a greater number of filled teeth was found among participants with a shorter nursing-home stay, and that a longer stay was associated with a greater number of missing teeth. Considering these results, it is possible that the number of general health problems increases in proportion to the duration of a resident’s nursing-home stay, with the result that oral care becomes less of a priority for the resident and caregivers alike. This shift in priorities might first affect the oral hygiene and then the oral health of nursing-home residents, finally resulting in the loss of remaining natural teeth. The risk of developing oral-health problems further increases among nursing-home residents who have fewer natural remaining teeth. Regrettably, dentist visits do not routinely occur in every nursing home, resulting in an unobserved deterioration of residents’ oral health. In this context, it should be noted that the deterioration of oral health not only affects chewing function, but also general health by aggravating systemic diseases. This, in turn, results in a greater risk of mortality [26, 28].

### Strengths and weaknesses of the study

One strength of this study is that all eligible participants who wanted to participate and gave informed consent were included, irrespective of their cognitive status and care needs. It is possible, however, those who chose to participate were more interested in their oral health than non-responders. It is also possible that some participants suspected they had acute dental problems and participated for this reason. It should be acknowledged that the study’s comprehensive medical, psychological and dental examinations were time-consuming; this undoubtedly deterred some people from taking part. Nevertheless, psychological examinations were performed first to reduce this bias. All other examinations were objective and were not dependent on the participation of the nursing-home resident. It should be noted that the general and dental health of the nursing-home residents are comparable to those found in other recent studies [4, 8–10, 12, 23], thus potentially enabling the results of this study to be generalised although it is local in character. In this context one should recognize that all study participants were comprehensively examined by dentists which cares for additional and valid information compared to retrospective or questionnaire studies. One weakness of the study is the manner of evaluation for the level of care required, which proved to be a relevant factor for oral hygiene and health. Use of the Barthel Index would have provided a more precise assessment of this factor and would have improved comparability with other studies. It should be noted that the coefficient of determination *R*^2^ is low in our regression analyses, indicating high variability of the dependent variables.

## Conclusion

Oral and denture hygiene and oral health remain inadequate among nursing-home residents, and the need for dental and denture-related treatment is acute. The most important risk factors for oral hygiene and health problems are poor general health, the presence of dementia, and motor impairment (apraxia). Because dentist visits to nursing homes are irregular and the consequences of diminished oral health conditions are potentially serious, implementation of a routinely performed oral health assessment tool by the care givers in nursing homes might be recommended. This should be kept in mind when planning oral-hygiene and health strategies in nursing homes.
